# Human Granzyme B Based Targeted Cytolytic Fusion Proteins

**DOI:** 10.3390/biomedicines6020072

**Published:** 2018-06-20

**Authors:** Precious Hlongwane, Neelakshi Mungra, Suresh Madheswaran, Olusiji A. Akinrinmade, Shivan Chetty, Stefan Barth

**Affiliations:** 1 Medical Biotechnology and Immunotherapy Unit, Institute of Infectious Disease and Molecular Medicine, Faculty of Health Sciences, University of Cape Town, Cape Town 7700, South Africa; hlnpre001@myuct.ac.za (P.H.); mngnee002@myuct.ac.za (N.M.); 2 South African Research Chair in Cancer Biotechnology, Department of Integrative Biomedical Sciences, Faculty of Health Sciences, University of Cape Town, Cape Town 7700, South Africa; mdhsur001@myuct.ac.za (S.M.); alex.akinrinmadex@gmail.com (O.A.A.)

**Keywords:** cancer immunotherapy, granzyme B (GrB), human cytolytic fusion proteins (hCFPs), immunotoxins (ITs)

## Abstract

Cancer immunotherapy aims to selectively target and kill tumor cells whilst limiting the damage to healthy tissues. Controlled delivery of plant, bacterial and human toxins or enzymes has been shown to promote the induction of apoptosis in cancerous cells. The 4th generation of targeted effectors are being designed to be as humanized as possible—a solution to the problem of immunogenicity encountered with existing generations. Granzymes are serine proteases which naturally function in humans as integral cytolytic effectors during the programmed cell death of cancerous and pathogen-infected cells. Secreted predominantly by cytotoxic T lymphocytes and natural killer cells, granzymes function mechanistically by caspase-dependent or caspase-independent pathways. These natural characteristics make granzymes one of the most promising human enzymes for use in the development of fusion protein-based targeted therapeutic strategies for various cancers. In this review, we explore research involving the use of granzymes as cytolytic effectors fused to antibody fragments as selective binding domains.

## 1. Introduction to Targeted Therapy and Humanization of Immunotoxins

An intervention that selectively repairs or kills cancerous cells, with no off-target related toxicity, represents the gold standard of targeted elimination of unwanted cells. One of the most promising approaches to date is based on protein engineering. Over the past 30 years, improvements in medical biotechnology have supported the development of four generations of therapeutic cell targeting ligand-toxin/enzyme named immunotoxins [1,2]. Immunotoxins (ITs) are bifunctional molecules consisting of a cell-specific binding ligand genetically fused or chemically conjugated to a cytotoxic component. The 1st and 2nd generations of ITs were produced by chemically conjugating native bacterial (*Pseudomonas aeruginosa* Exotoxin A) or plant toxins (ricin and gelonin) to full-length murine antibodies [3,4,5]. However, several challenges were encountered, ranging from poor specificity and stability to heterogeneity. Fortunately, through the use of recombinant DNA technology, the 3rd generation of ITs have been introduced, consisting primarily of a single-chain variable fragment (scFv) directly linked to bacterial toxins [6,7]. Such recombinant ITs exhibit better efficacy and tumor penetration. Though a step in the right direction, immune rejection remains a debilitating problem. Hence, this has led to the rise of the latest and 4th generation of ITs termed as human cytolytic fusion proteins (hCFPs). These hCFPs are designed by replacing existing toxins with human pro-apoptotic proteins capable of inducing cell death [8,9,10,11,12,13,14].

Therefore, the combined specificity of targeting the human ligand and the apoptosis-inducing effector protein offers a palpable impact, including reduced immunogenicity and toxicity, high selectivity and increased tumor penetration [15]. Of the various human apoptosis-inducing enzymes, granzyme B (GrB)-mediated apoptosis of target cells has been clinically associated with improved patient outcomes for various types of cancers. Human GrB belongs to a family of five serine proteases called granzymes, which were discovered in the cytoplasmic granules of natural killer (NK) cells and cytotoxic T lymphocytes (CTLs) in the 1980s [16,17,18]. Since their discovery, scientists have attempted to investigate the major role that they play in the destruction of malignant or virus-infected cells [19]. Additionally, in furtherance of the development of hCFPs, researchers have also explored the apoptosis-inducing mechanisms of GrB [20,21,22]. On that account, this review represents an updated understanding of the importance of GrB in the establishment of recent hCFPs.

## 2. Granzyme B and Its Anti-Tumor Activity

GrB (32-kDa) is reported to be the most potent of all the human granzymes produced by CTLs [22]. Due to its cytotoxic nature, it is expressed as an inactive prepro-enzyme and becomes functional by the removal of two pro-peptide residues (Gly-Glu dipeptide from its N-terminus) by lysosomal dipeptidyl peptidase I/cathepsin C [23]. Its biological activity during a CTL or NK cell-mediated immune response is dependent upon: (i) co-release with pore forming proteins called perforin towards target cells at intercellular spaces called immunological synapses [21,24]; (ii) successful entry into the cytosol of the cell (an event still broadly debated and hypothesized to be mediated by perforin, either through the formation of holes in the cell membrane or through destabilization of the ionic gradient to allow pore-formation in endosomal vesicles [25]); (iii) activation of several pro-apoptotic pathways by proteolytically attacking several intracellular protein substrates.

While up to about 300 intracellular proteins have been identified in humans as potential GrB substrates [22], only a few have been confirmed to be related to GrB-mediated apoptosis. For instance, the activation of multiple caspase family members (–3, –6, –7, –8, –9, –10) and cleavage of BH3-only pro-apoptotic protein (Bid) are well demonstrated in literature [26]. A nuclear pro-apoptotic pathway has also been reported for human GrB and involves cleavage of cell cycle regulatory proteins and/or kinase cell division cycle (CDC) activation. The potential of GrB to directly trigger post-caspase cytoplasmic apoptotic death pathway has also been described [23]. Therefore, the ability to activate multiple pro-apoptosis inducing pathways (including the induction of DNA fragmentation) in target cells, is what makes the development of GrB-based fusion proteins an attractive solution for cancer therapy.

Although highly efficient in its apoptosis-inducing mechanisms, the design of granzyme-based targeted therapeutics suffers an important hurdle: GrB possesses a number of basic amino acids on its surface, which results in a high isoelectric point [27]. This culminates in the unspecific binding or uptake of GrB by negatively charged cells. Reduced specificity increases the probability of immunogenicity, thus decreasing the therapeutic potential of the immunotoxin. Additionally, to prevent CTLs and by-standing cells from GrB mediated-cleavage, its enzymatic activity is tightly controlled by the presence of inhibitor Serpin B9 (PI-9, also known as proteinase inhibitor 9). PI-9 first forms a reversible Michaelis-like complex with GrB and then covalently binds to it with a stoichiometric ratio of 1:1 [28]. The up-regulation of PI-9 is useful for health cells, however tumor cells evade destruction by producing this inhibitor and making GrB-based targeted therapies ineffective.

To mitigate these shortcomings, continuous innovation in the design of GrB-based cytolytic fusion proteins has enabled steadily improved performance. The details of these are outlined in the following section.

### Granzyme B in Targeted Therapy

**Figure 1:**
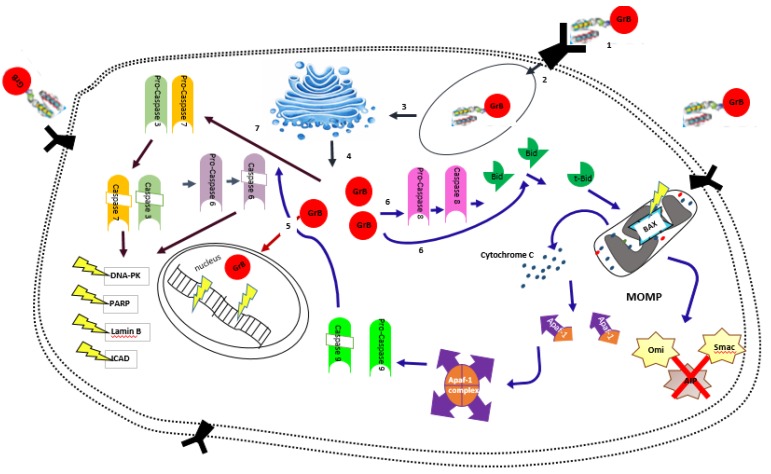
Mechanism of action for the targeted delivery of GrB to the tumor cell. (1) Binding of GrB hCFP to receptor on cell membrane; (2) Take up of hCFP into the cell; (3) Routing and processing in ER and Golgi leading to endosomal escape of GrB; (4) Active GrB released into the cytosol; (5) Direct cleavage of nuclear machinery; (6) Initiation of the mitochondrial/caspase independent pathway. GrB cleaves procaspase-8 leading to caspase-8 activation. Caspase-8 cleaves the BH3 interacting domain death antagonist (BID) leading to the activation of truncated Bid (t-Bid). T-Bid translocates into the mitochondria and activates pro-apoptolyic protein BAX. This leads to mitochondrial outer membrane permealization (MOMP) and release of cytochrome C, Smac/DIABLO and OMI/HTRA2 which promote the blocking of inhibitor of apoptosis protein (IAP). Cytochrome C binds to the apoptotic protease activating factor (APAF-1) in the presence of dATP, leading to the formation of the apotosome which activates procaspase 9. Activated caspase-9 cleaves procaspase-3 and -7; (7) Initiation of the caspase dependent pathway. GrB directly cleaves procaspase-3 and -7 leading to the activation of the executioner caspases -3 and -7. These caspases cleave procaspase-6 leading to activated caspase-6. Caspase-3, -6 and -7 cleave downstream substrates such as PARP (poly(ADP-ribose) polymerase), DNA-PK (DNA-dependent protein kinase), Lamin B and ICAD (inhibitor of caspase activated DNase).

The use of GrB in targeted therapy was pioneered simultaneously by the laboratories of Michael G. Rosenblum (M.D Anderson Cancer Center, Houston, Texas, USA) and Stefan Barth (IDM, Faculty of Health Sciences, University of Cape Town, South Africa) about a decade ago [29]. This novel concept involved the genetic fusion of an antibody moiety or derivative of a natural ligand targeting a surface protein or receptor on a diseased cell to the carboxyl end of GrB. Corresponding fusion proteins named immunoproteases allowed the selective ligand-mediated uptake and delivery of GrB into the target cells in the absence of perforin (Figure 1). Since then, a series of GrB-based hCFPs targeting different surface proteins on target cells have been developed for various diseases (Table 1). For instance, a GrB-H22(scFv) fusion protein has been shown to be effective in the killing of CD64+ U937 cells (lymphoblasts from human lung) and cells from acute myeloid leukaemia patients expressing CD64 as a surface receptor [30]. A significant activation of caspase-3 in the lysate of GrB-targeted treated cells when compared with the untreated control confirmed the potential of the GrB-H22(scFv) fusion protein to initiate apoptosis in target cells. Using a similar approach, early studies from the group of Rosenblum also demonstrated the therapeutic potential of GrB-based constructs. A GrB fusion to an anti-melanoma single chain antibody fragment (scFvMEL) targeting human A375-M melanoma cells induced apoptosis 8 h after treatment. Results showed both cleavage of caspase-3 and release of cytochrome C from the mitochondria into the cytosolic compartment as apoptosis pathways were mediated by the GrB-scFvMEL construct [31]. Consequently, several GrB-based cytolytic fusion proteins have been developed and evaluated against various carcinomas [31,32,33,34]. In recent years, newer versions of GrB-based hCFPs have emerged against existing clinical limitations and are reviewed below.

## 3. Reducing the Off-Target Toxicity of Granzyme B for Future Targeted Therapies

Over the years, clinical limitations of GrB-based constructs have driven research into the development of highly innovative constructs that can efficiently deliver active/cleaved GrB into the cytosol of target cells following systemic application without any therapeutic limitation. Below, we document data on highly improved versions of GrB-based hCFPs produced by Kurschus and Bird in the last decade (Table 2).

Off-target toxicity and bioavailability of immunoconjugates are important topics in the clinical evaluation of targeted therapies. In general, the lack of disease specific surface target is the key reason for off-target toxicity in targeted therapy. The other possibility is the ability of immunoconjugates (recombinant GrB) to bind to various types of receptor in addition to the receptor of interest, say for example, GrB has the ability to bind and activate PAR-1 receptor and induce neuronal apoptosis in vitro and in vivo [38]. Jabulowsky stated that recombinant GrB binds to non-target tissues by forming electrostatic interactions between the heparin-binding motifs RKAKRTR and KKTMKR of GrB with glucosaminoglycans found on the cell surface of these non-target tissues. The author then intended to replace the positively charged amino acids at 116, 120, 122, 241, 242, 245 and 246 located in the heparin binding motifs to Alanine, and generated mutant GrBcs which show similar enzymatic activity and cytotoxicity, and reduced unspecific binding to non-targeted tissues, as compared to wild type GrB [39]. This will eventually not only reduce off-target toxicity, but also increase the bioavailability (increase the amount) of GrB in targeted cells, as non-specific binding could limit the amount of protein available for tumor-specific cell killing [39]. In natural GrB that are exocytosed by NK cells and T cells, the positively charged amino acids in heparin binding motifs and other motifs form complexes with negatively charged sulphate proteoglycan serglycin (SG), resulting in the shielding of its positively charged surface [39,40]. Thus, the use of GrB-SG complex as a therapeutic moiety can be used to attenuate the problem of off-target toxicity. Another method would be to encapsulate the recombinant GrB with another protein to prevent non-specific binding of GrB.

Moreover, as mentioned previously, endogenous GrB is in its inactive form when it bears the N-terminal Gly-Glu dipeptide, which is cleaved off by dipeptidyl peptidase cathepsin C (CatC) in lytic granules inside immune cell and frees the newly N-terminal Ile 16 residue to form a salt bridge with Asp 194 [23,41]. The resulting conformational change enables the simultaneous generation of an oxyanion hole and maturation of the active-site S1 pocket. This active form of endogenous GrB is released from lytic granules and has the ability to kill any target cell it enters. Based on this fundamental concept, scientists started to use the inactive form of GrB by filling the free NH_2_ terminus with an N-terminal inhibitory peptide (SUMO1 peptide) which can be selectively removed by cognate proteases, called sentrin-specific proteases (SENPs). These SENPs are highly expressed in tumor cells and can be used to activate GrB’s catalytic function. Such engineered GrB molecules would require both a surface-bound antigen and an intracellular protease to trigger target-cell apoptosis, thereby increasing targeting specificity and the safety profile of GrB-based therapeutics [42].

## 4. Overcoming Natural Granzyme Inhibitors

Cytotoxic T Lymphocytes (CTLs) and Natural Killer (NK) cells are the main immune effector cells responsible for killing infected and transformed cells. They do this by delivering cytotoxic granules (Granzyme B) stored in lysosome-related organelles (LROs), into the targeted cell cytoplasm [43]. The LRO fuses with the plasma membranes and releases GrB into a synapse or cleft between the effector and target cell [44]. Synaptic release of GrB from CTLs may cause fratricide and suicide of CTL/NK cells. This happens when GrB leaks back into the immune cell. In addition, secreted GrB can be endocytosed by CTLs or bystander cells and be released in the cytoplasm after endolysosomic stress [45], culminating in fratricide and/or suicide. To avoid this self-killing mechanism and to protect CTLs and NK cells from leaked GrB, the cell has its own natural nucleocytoplasmic protease inhibitor PI-9 which inactivates GrB proteases by acting as suicide substrates [46] through the formation of a 1:1 complex [47]. The release of GrB into cytosol in CTL/NK cells is tightly controlled and is not accidental. Here, the removal of immune cells that are under constant stress conditions becomes essential; repeated stimulation of activated CTLs and NK cells will lead to LRO damage and lysosomal membrane permeabilization, which releases the lysosomal contents (GrB) into the cytosol. During this process, the protective mechanism of PI-9 in CTLs is aborted and the cell undergoes activation-induced cell death (AICD). Thus, the survival of CTLs and NK cells depends on the balance between the amount of GrB released and the level of active PI-9 in the cytosol [43], which varies when cell is under stress.

Certain cancers of the lung [48], breast [49] and prostate [50] have increased levels of PI-9 in the cytosol that evade immune destruction, thereby hampering the therapeutic use of recombinant wild type GrB as a cytotoxic agent [37]. However, the findings from some studies beg to differ; in some, the endogenous presence of PI-9 has had no effect on the cytotoxic effect of their constructs [51,52]. In hypoxic conditions, cancer cells selectively degrade GrB by forming a fusion of amphisome (the fusion of enlarged endosomes containing GrB and autophagosomes) and lysosome [53]. To overcome this challenge, seven variants (R28A, R28E, R28K, R201A, R201E, R201K, K27A) of GrB were engineered by the Barth and Carloni group to be insensitive to PI-9 (Table 3). Their work was based primarily on using computational alanine scanning mutagenesis (CASM) to identify residues on GrB that might be crucial for its interaction with PI-9. Thereafter, some of these variants were selected and molecular dynamics simulations were carried out to determine the effect of these variants on the stability of the GrB-PI-9 complex in aqueous solution. Interestingly, the R201K variant was the most promising and resistant, as it maintained the same enzymatic activity in the presence and absence of recombinant PI-9 in our in vitro studies [54].

More recently, Rosenblum and colleagues also reported similar mutations on the GrB primary sequence which reduced binding to PI-9. Two double mutants termed EA “K27E and R28A” and LA “K27L and R28A” respectively demonstrated insensitivity towards PI-9 without any effect on enzymatic activity [41]. Of note is that the enzymatic activity of EA was reported to remain over 40% in the presence of 50% human serum when compared to unmodified GrB-fused to VEGF_121_, whose enzymatic activity declined to less than 10% activity over the same time period [41].

## 5. Effective Cytosolic Delivery of Granzyme B to Target Cells: A Major Bottleneck

Lastly, a major limiting factor to the activity of existing GrB-based fusion proteins, is the effective translocation and delivery of the lethal GrB molecules from the endosomes to the cytosol of the target cell. In its natural state, GrB is secreted along with perforin, an endosomolytic agent that facilitates the cytosolic release of GrB from endocytic compartments [55]. However, the application of GrB as a human lead enzyme has led researchers to consider alternative methods to perforin-mediated cytosolic release. For instance, to allow the cytosolic uptake of GrB fusion proteins targeting EGFR/ErbB2, Dalken et al. used chloroquine to raise the pH value in endosomes and lysosomes, thereby culminating in the osmotic rupture of these vesicles [32]. In another study, the translocation domain of *Pseudomonas* exotoxin A (PE-II), was fused to a HER2(scFv) and GrB, demonstrating the effective killing of HER2-overexpressing cells both in vitro and in nude mice [56]. While acknowledging that this method induces tumor growth inhibition, Kurschus and colleague raise the possibility of immune rejection [57]. An alternative approach to consider would be the use of sophisticated protein transduction domains (PTD) flanked with cleavable adapters, which would allow the specific and efficient transport of human enzymes into the cytosol of target cells [58,59]. Further investigations are still required to determine whether other endosomolytic agents or adapter sequences can be used to increase the cytotoxic activity of GrB-based hCFPs.

## 6. Conclusions

Whilst GrB as the natural human apoptosis-inducing enzyme has clinical potential when delivered as a ligand-specific fusion protein, strict inhibition by PI-9 may cause resistance to therapy. As shown by the Barth and Carloni group [54], computational approaches can now be used to study enzyme-substrate interactions to greater depth. These might be a useful approach to consider for finding new mutations that might reduce PI-9 binding to GrB. Moreover, for effective translation of scientific research from bench to bedside, further in vitro and in vivo studies are urgently needed to assess the activity, efficacy and safety of promising mutant candidates described in this review. Such studies form the basis for the continued optimization of this effective human enzyme in the future of cancer therapy.

## Figures and Tables

**Table 1:** Granzyme B based human Cytolytic Fusion Proteins (hCFPs) resistant to serpin B9 inhibition, targeting surface proteins expressed in various cancers.

Construct	Disease *	Target	Cell Line	P19 Expression in Cell Line	Cytotoxicity	Reference
GrB (wt)-H22(scFv) and GrBR201K-H22(scFv)	CMML	CD64+	Cells from AMML and CMML patients.	Yes	Not specified	[35]
			CD64+ HL60	No	4–7 nM	
GrB (wt)-ki4(scFv) and GrBR201K-Ki4(scFv)	cHL	CD30+	L428	Yes		[12]
			L540cy	No		
GrBR201K-scFv1711	Epidermoid cancer cells	EGFR+	A431	Yes	133.3 nM	[36]
			RD target cells	Yes	21.1 nM	
GrBR201K-αEpCAM(scFv)	TNBC	EpCAM+	MDA-MB-231	Yes	N/A	[37]
			MDA-MB-468	yes	221 nM	
			MDA-MB-453	No	307 nM	

* AMML, Acute myelomonocytic leukemia; CMML, Chronic myelomonocytic leukemia; cHL, Classical Hodgkin’s lymphoma; TNBC, Triple-negative breast cancer.

**Table 2:** Granzyme B mutants for improved specific binding and reduced off-target toxicity.

Granzyme B Variant	Mutation	Implication of Mutation	Result	Reference
GzmB^FacD^	The kktmrkry sequence at the C-terminus was replaced with the acidic peptide DSVLA derived from human complement factor D	This sequence motif is not positively charged and should have little immunogenic potential because complement factor D occurs at relatively high levels in human plasma	Binding to HL60 cells was completely abolished	[21]
GzmB^KD^	The region around K127 and K131 is known to function as a heparin binding site in thrombin. To stabilize this, both lysines were replaced with aspartate residues	Reduced HS binding	Reduced binding to HL60 cells compared to wild type GrB	[21]
GzmB^KD-FacD^	Double mutant consisting with aspartate replacement at position K127 and K131 and the acidic C-terminal peptide DSVLA	Combined effect of mutation	The binding and internalization efficiency was completely abolished	[21]
cs1	Arginine in position 110, 114 and 116 (R110, R114, and R116) replaced with alanine. Constitutes an altered classical GAG-binding motif	Most proteins bind GAG. This is dependent on electrostatic interaction between the positively and negatively charged cells. Mutation in this region alters binding of GrB to negatively charged cells	Reduced cytotoxic activity. 20-fold less cytotoxic compared to wild type GrB. Abolished binding to Heparin region	[27]
cs2	Lysine in position 239, 240, 243 and 244 (K239, K240, K243, and K244) replaced with alanine. Constitutes an altered C-terminal helix	Amphipathic C-terminal helix that has paired basic residues that bind GAGs. Mutation in this region alters binding of GrB to negatively charged cells.	Reduced cytotoxic activity. 2.5-fold less cytotoxic compared to wild type GrB. Reduced binding to Heparin region	[27]
cs1+2	Combined mutation of cs1 and cs2	Combined mutation of cs1 and cs3	Reduced cytotoxic activity. 20-fold less cytotoxic compared to wild type GrB. Abolished binding to Heparin region	[27]

**Table 3:** Granzyme B variants to improve cytotoxicity and bypass serpin B9 inhibition.

Granzyme B Variant	Mutation	Implication of Mutation	References
R28A	Substitution of Arginine residue with Alanine (constitutes a neutral charge at position 28)	In the presence of PI-9 the GrBR28A mutant contains 54% activity	[54]
R28E	Substitution of Arginine residue with Glutamate (constitutes an opposite charge at position 28)	In the presence of PI-9 the GrBR28E mutant contains 25% activity	[54]
R28K	Substitution of Arginine residue with Lysine (constitutes an identical charge at position 28)	In the presence of PI-9, the GrBR28K and mutants retained 76% of their original activity	[54]
R201A	Substitution of Arginine residue with Alanine (constitutes a neutral charge at position 201)	In the presence of PI-9, the GrBR201A mutants retained 46% of their original activity	[54]
R201E	Substitution of Arginine residue with Glutamate (constitutes an opposite charge at position 201)	No activity in the presence of PI-9	[54]
R201K	Substitution of Arginine residue with Lysine (constitutes an identical charge at position 28)	In the presence of PI-9, the GrBR201K mutant retained 94% of its activity	[54]
K27A	Substitution of Lysine residue with Alanine (constitutes a neutral charge at position 27)	Insensitive to P1-9 activity and K27A mutant showed a marked decrease in the ability to bind and cleave a substrate (substrate 3) containing P9 residues	[54]
R28A & R201A	Double mutant; Arginine replaced with Alanine at position 28 and 201	In the presence of PI-9 the double mutant contains 0.5% activity	[54]
K27E & R28A (EA)	Double mutant; Lysine replaced with Glutamate at position 27 and Arginine replaced with Alanine at position 28	In the presence of 50% human serum, the enzymatic activity of EA remained over 40% over 24 h	[41]
K27L & R28A (LA)	Double mutant; Lysine replaced with Leucine at position 27 and Arginine replaced with Alanine at position 28	LA double mutant appeared to behave intermediate to the wild-type protein (GrB/VEGF_121_) and the EA construct	[41]
